# Nano-Neurotheranostics: Impact of Nanoparticles on Neural Dysfunctions and Strategies to Reduce Toxicity for Improved Efficacy

**DOI:** 10.3389/fphar.2021.612692

**Published:** 2021-03-26

**Authors:** Chiluka Vinod, Srikanta Jena

**Affiliations:** ^1^ Department of Biological Sciences, School of Applied Sciences, KIIT University, Bhubaneswar, India; ^2^ Department of Zoology, School of Life Sciences, Ravenshaw University, Cuttack, India

**Keywords:** nanotheranostics, imaging, blood brain barrier, neurotherapy, toxicity

## Abstract

Nanotheranostics is one of the emerging research areas in the field of nanobiotechnology offering exciting promises for diagnosis, bio-separation, imaging mechanisms, hyperthermia, phototherapy, chemotherapy, drug delivery, gene delivery, among other uses. The major criteria for any nanotheranostic-materials is 1) to interact with proteins and cells without meddling with their basic activities, 2) to maintain their physical properties after surface modifications and 3) must be nontoxic. One of the challenging targets for nanotheranostics is the nervous system with major hindrances from the neurovascular units, the functional units of blood-brain barrier. As blood-brain barrier is crucial for protecting the CNS from toxins and metabolic fluctuations, most of the synthetic nanomaterials cannot pass through this barrier making it difficult for diagnosing or targeting the cells. Biodegradable nanoparticles show a promising role in this aspect. Certain neural pathologies have compromised barrier creating a path for most of the nanoparticles to enter into the cells. However, such carriers may pose a risk of side effects to non-neural tissues and their toxicity needs to be elucidated at preclinical levels. This article reviews about the different types of nanotheranostic strategies applied in nervous dysfunctions. Further, the side effects of these carriers are reviewed and appropriate methods to test the toxicity of such nano-carriers are suggested to improve the effectiveness of nano-carrier based diagnosis and treatments.

## Introduction

One of the important advancements in nanotechnology is the evolution of dual-purpose strategies which can be used for both therapy and diagnosis. This dual nanomolecules in other terms is also known as theranostics. This term indicates a nanotherapeutic system which is integrated for delivery, diagnosis, and can monitor relative responses (Warner, 2004).

One of the emerging research in the field of drug delivery is the combination of biochemistry and molecular biology with nanotechnology, also known as nanobiotechnology. Nanoparticles have attained significant attention due to their versatility and potential applications (Gao et al., 2009). Thus they are considered as one of the most promising methods to treat cancer and many other diseases. Compared to many other synthetic drug formulations, nanomaterials are considered as most feasible or biocompatible in the field of medicine, or in studying few vital mechanisms in biological organisms (Chenthamara et al., 2019). Hence applying nanotechnology in these areas necessitates various settings to be considered. Primarily, devising the nanomaterials be required to interact with the signalling components such as proteins and other biomolecules in a cell without impeding with their cellular function. Furthermore, the physical properties of nanomaterials have to remain unaltered after superficial modifications. Lastly, it is essential to be nontoxic (Solanki et al., 2008).

## Nanoparticles Used in Physiological Applications

Of all the contrast agents used as theranostics, the majority are used in imaging mechanisms. The most applicable imaging mechanism among others is magnetic resonance imaging (MRI). Most of the research in imaging is hence attributed to MRI in which magnetic particles are used as the contrast agents. These nanoparticles in MRI serves the purpose of marking the diseased tissues and/or cells in contrast to healthy tissues and/or cells. Particles with good magnetic properties such as silver, iron oxide, gadolinium, gold and other metals are being investigated at several levels to find a suitable nanoparticle with the least toxicological effects. Further, the selection of nanomaterials and functionals aspects with their allied polymers/materials has to be studied to confirm their biocompatibility and biodegradability profile (Li et al., 2004; Morel et al., 2008; Souza et al., 2008). A brief overview of general nanoparticles and their applications is mentioned in Table 1.

**TABLE 1:** An overview of nanoparticles and their applications in nervous system.

Nanoparticle	Applications	References
Carbon Nano Tubes	Diagnosis, DNA and drug delivery	Karthivashan et al. (2018), Aguilar Cosme et al. (2019)
Gold	Diagnosis, tumour targeting and PTT	Perets et al. (2019), Spinelli et al. (2019)
Iron oxide	docetaxel Targeting, MRI and therapy	Tomitaka et al. (2015), Saesoo et al. (2018)
Manganese oxide	MRI plus RNA delivery	Bae et al. (2011), McDonagh et al. (2016)
Silica	Drug carrier, X-ray/CT imaging, Photodynamic therapy	Song et al. (2017), Turan et al. (2019)
Quantum Dots	Imaging, therapy and sensing	Paris-Robidas et al. (2016), Matea et al. (2017)

### Ironoxide Nanoparticles

Among the nanoparticles with superparamagnetic abilities, Iron oxide nanoparticles (IONPs) gets the highest choice as contrast agents because of their biocompatibility and low cost, enabling easy availability to a wide range of research groups. The common form of IONPs used will either be a magnetite or hematite. The addition of several other polymers or macromolecules further modify the surface making the IONPs more stable and in addition enhance the efficacy of IONP-based agents for diverse applications. These additions on the surface of IONPs made significant advancements in diagnosis with therapy with major advancements been found in MRI and drug delivery purposes (Kohler et al., 2005).

Since the discovery of these theranostic additions, majority of biological experiments in aspects of imaging and delivery concentrated on the preparation of an all-in one target-cell-specific IONPs using diverse delivery mechanisms such as siRNA, plasmid delivery, etc. In addition to these the magnetic nano-vectors are employed to exploit certain mechanisms of cell such as the cell transcytosis intracellular trafficking (Lee et al., 2007; Lee et al., 2009; Veiseh et al., 2011).

In an experiment, PEGylated superparamagnetic iron oxide (SPIO) nanoparticles were conjugated with anticancer drug and the other distal end was conjugated with tumour-targeting ligands. These wormlike polymer vesicles loaded with DOX and SPIONs, prepared by hetero-bifunctional triblock copolymer R (methoxy or FA)-PEG114-PLAx-PEG46-acrylate via a double emulsion technique was found to have higher stability and multifunctional with higher ability to targeted cancer therapy and ultrasensitive MRI (Jain et al., 2008; Yang et al., 2011).

For treating the neurological disorders nanoconjugates were prepared by using dopamine on IONPs and are encapsulated into human serum albumin (HSA) matrices. As mentioned earlier these conjugated nanocomposites can act as all-in one platform where one can use them in diverse applications such as MRI, down-conversion fluorescence (FL) imaging, upconversion luminescence (UCL) and magnetic drug delivery for *in vivo* and *in vitro* use (Xu et al., 2011))

Carbon nanotubes (CNTs) are cylindrical structures with a single or multilayered cylinders with unique mechanical and electronic properties. The common applications of CNTs are drug or gene delivery and imaging thermal ablation. Although many researchers tried to explore the mechanism of how these CNTs are efficiently uptaken by cells, the exact mechanism is obscure. These are the most common templates used in drug delivery for loading different active agents. They have attained theranostic criteria due to the strong optical absorbance at near-infrared (NIR) photothermal ablation therapy. In addition to photothermal ablation therapy, they were also reported to be used for photoacoustic imaging. In a report, both thermal ablation and photoacoustic tomography were demonstrated to reduce the tumour. (Krishna et al., 2010).

Quantum dots (QDs) are the elements belonging to transition groups which are inorganic and act as semiconductor fluorophores. The primary application of QDs in biology is imaging and secondarily for drug delivery. The toxicological profile of QDs is higher due to which they have been subjected to less research. However, nowadays QDs are less used for imaging purposes (especially *in vitro*) and mostly used for therapeutics. In majority of the cancers, the DOX delivery path was sensed and the targets were observed by imaging using fluorescence imaging with QD (Matea et al., 2017).

Gold-based nanoshells with properties of magnetism and optics are widely used for theranostics in neurophysiology. The iron-oxide nanoshells (which are superparamagnetic in nature) were gold coated and are conjugated with the targeting agent. These conjugates of gold-based nanoshells and the drugs were investigated for use in neck and head cancer. In addition several nanocages of gold-silver combined with yb-2,4-dimethoxyhematoporphyrin were shown to have multifunctional infrared luminescence detection abilities, and also were employed for photothermolysis and photosensitisation. One of the successful conjugate models was nanocomposition of silica-modified gold nanorods combined with folic-acid which was used for photothermal therapy and dual-mode radiation (X-ray/CT imaging-guided) (Huang et al., 2011). In nanomedicine, it is normally used as a coating material to provide or avoid different nanoparticle characteristics (Yang et al., 2012).

## Nanoparticles Employed in Neural Dysfunction

According to WHO report brain disorders are responsible for nearly 12% of death in the world (WHO, 2006). The cerebro-vascular dysfunctions take the major proportion (about 85%) among them. The major reason is not the activity of the respective drug(s) but being unable to cross the blood-brain barrier (BBB). Hence overcoming the BBB needs to be the first criteria while preparing a therapeutic (Guiot et al., 2016).

The last decade has been effective in overcoming the BBB through several physical and chemical approaches. Majority of the research and reviews on this topic have been published during this period. Few efficient methods were discussed below.

**FIGURE 1:**
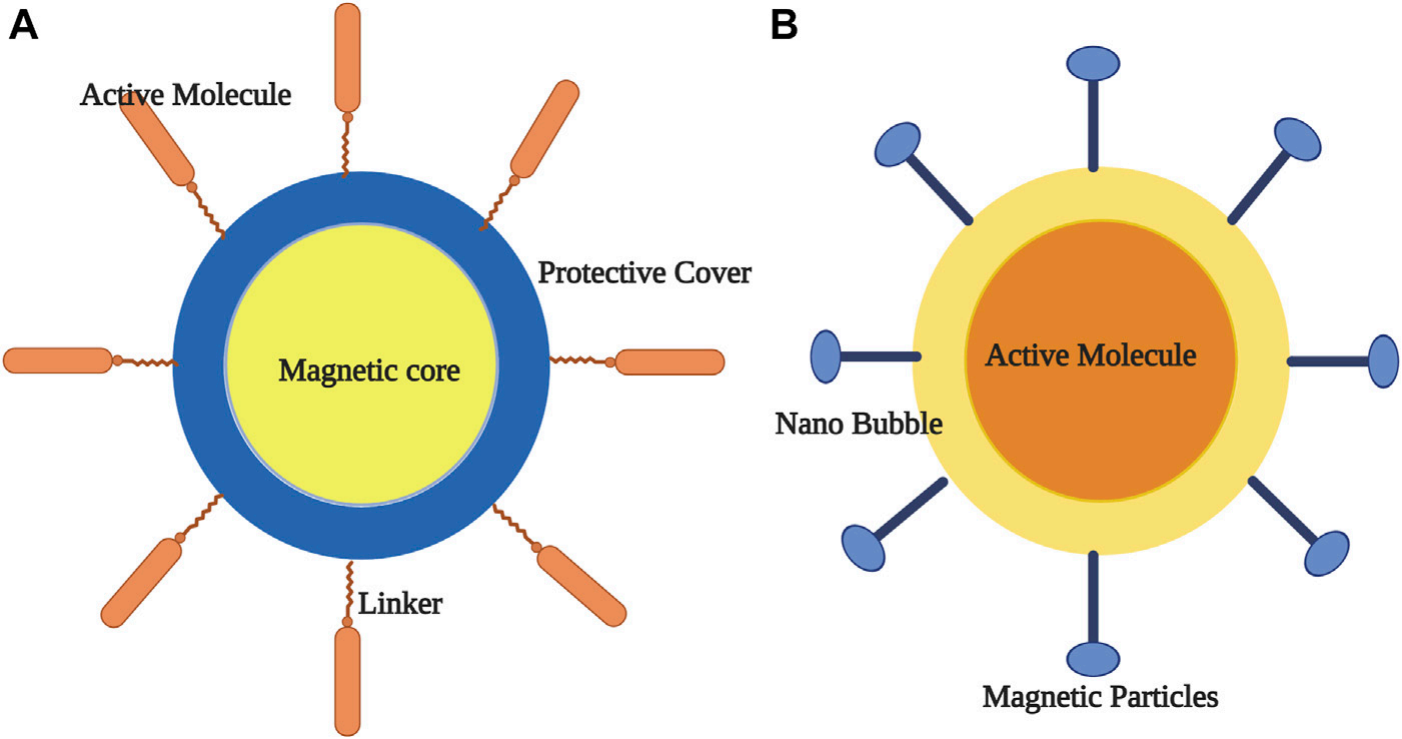
Methods of preparation of nanoparticles.

**FIGURE 2:**
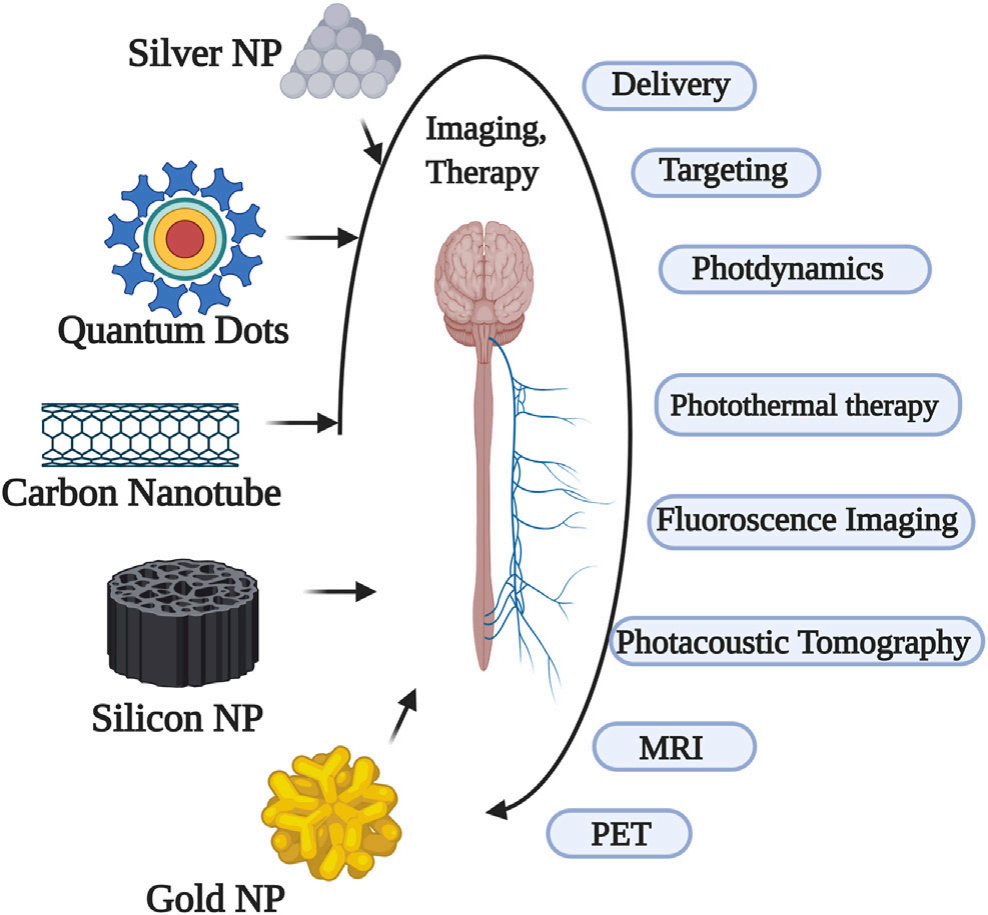
Various nanoparticles and their applications in nervous system.

Smart nanoparticles (SNPs) with magnetic properties (SMNPs) are proven to be effective in drug delivery across BBB as they are efficient in magnetoporation in BBB endothelium (Lakshmanan et al., 2014.). The magnetic forces are dependent on size, coating and shape of the nanoparticles. It is found that the magnetic targeting is most effective in body surfaces where the blood flow is slower (Chertok et al., 2007). Colloidal ferrofluids with a biocompatible polymeric layer coating has been reported to improve the stability of SMNPs (Figure 1A). In a set of experiments when Dextran coated MNPs were targeted into hippocampus through osmotin loaded delivery resulted in reduced protein accumulation and memory improvement in an Alzheimer’s disease (AD) rats (Faustino et al., 2017). Similar results were also reported using Uncoated Fe3O4 (Umarao et al., 2016). In several studies using heat-sensitive capsaicin receptors along with MNPs into the neurons or specific regions of brain, notably resulted in modulation of neural or the brain-areas activity respectively which provided an insight for deep brain stimulations, a potential application needed for treating various CNS diseases (Figure 2) (Chen et al., 2015; Munshi et al., 2017; Tay and Carlo, 2017). In all the above cases the use of magnetic resonance imaging were proposed to observe the real-time imaging and driving of MNPs in deeper tissues. However, the drawback with this method is the majority of the traditional MRI scanners needs customization for particle driving with an additional challenge of driving a large number of MNPs to a precise area in the brain (Figure 2).

Carbon NPs (CNP) were proven to be effective carriers of antioxidant enzymes in cases of traumatic brain injuries (TBI) where a tremendous increase in Reactive oxygen species (ROS) and Reactive Nitrogen Species (RNS) species were reported (Reddy and Labhasetwar, 2009; Singhal et al., 2013). In a report, the use of multi welled CNPs conjugated with Pittsburgh Compound B derivates such as Gd (L2) or Gd (L3) also was shown to cross the BBB efficiently (Costa et al., 2018). In addition use of polyamidoamine dendrimer derivative, NPs also showed robust drug delivery and promising recovery from TBI (Nance et al., 2016).

Solid Lipid Nanoparticles (SLNPs) were able to deliver 3,5-dioctanoyl-5-fluoro-2-deoxyuridine drug two fold efficiently compared to free fluoro-2-deoxyuridine as the SLNPs were diffused along the blood capillaries retaining the drug in the brain for longer periods (Martins et al., 2012).

PBCA (polybutyl cyanoacrylate) NPs (of size 87–464 nm) coated with Apo-E-bound polysorbate 80 are demonstrated to be actively taken up by endothelial cells of the brain as they are recognized as low-density lipoproteins (LDL) mediated by receptor based-endocytosis (Voigt et al., 2014).

Polyfluorene-chitosan conjugates were also reported to be efficient in crossing the BBB with an additional ability to preclude the aggregations of Aβ1-40 thus interfering with AD like pathologies (Roy Chowdhury et al., 2018).

PEG coated Gold (Au) NPs when tagged with a transactivator of transcription (TAT) peptide were shown to cross BBB through passive diffusion and their accumulations were observed in both endothelial cells and the brain tumours (Cheng et al., 2014)

Silica NPs (of sizes 25–40 nm) loaded with DOX have been demonstrated to be one of the promising strategies to treat brain cancers (Mo et al., 2016; Song et al., 2017)

However, Carbon Dots (CDs) display more theranostic values than the Silica NPs owing to their innate fluorescence abilities and non-toxic properties (Figure 2). At a size of 2.6 nm and 51% quantum yield these NPs conjugated with cationic PEI were reported to fit within the tight junctions of BBB and cross it effectively (Lu et al., 2016).

Extracellular vesicles (EVs) are one of the intrinsically available carriers which are potential delivering agents due to their ability to cross BBB easily and also takes part in functional aspects of the cell. Extensive studies have implicated that the EVs play a major role from different regions of neurons, for eg: EVs from dendritic cells are observed to have remyelination abilities as observed in a model of multiple sclerosis (MS), EVs from Schwann cells were reported to possess axonal regeneration abilities, EVS from oligodendrocytes were crucial for neuronal integrity as they take part in releasing neurotransmitters (Fuster-Matanzo et al., 2015). This feature was exploited by many researchers in treating curricula of diseases such as cancer, experimental autoimmune encephalomyelitis, AD, PD, etc (Zhuang et al., 2011; Cooper et al., 2014). This opened up scopes for treatment of various neurological disorders.

The size and shapes of nanoparticles also were demonstrated to have an effect on delivery into the brain. Particles of size beyond 400 nm were not considered for delivery purpose. Particles of 200 nm or more usually are prone to reticulo-endothelial clearance in liver and spleen. On the other hand, particles of 10 nm or lesser are rapidly filtered by kidneys (Blanco et al.,2015). However, some researchers attained success even with a lower size of NPs with certain modifications. Similarly, rod shaped or cylindrical nanoparticles were found to be more effective in drug delivery compared to spherical nanoparticles (Barua et al., 2013; Kolhar et al., 2013). Interestingly, only two routes of endocytosis are reported to favour the transport of NPs across the BBB, 1) clathrin-mediated endocytosis which can accommodate 200 nm nanoparticle and 2) Caveolin mediated endocytosis which can take upto 80 nm nanoparticles (Cena and Jativa, 2018). An overview of effective nanoparticle size, shape and method is being briefed in Table 2.

**TABLE 2:** Recent works demonstrating Effective Nanoparticle sizes to cross BBB.

Nanoparticle Used	Effective Size and shape to cross BBB	References
Carboxylated polystyrene	200 nm Spheres	Nowak et al. (2020)
Carboxylated polystyrene	200 nm rods. (2x efficient than spheres)	Nowak et al. (2020)
Cobalt-ferrite NPs	30 nm	Pardo et al. (2020)
Fluorosceine conjugated Gold NPs	2 nm (Demonstrated in BBB spheroids)	Sokolova et al. (2020)
Focused Ultrasound targeting of Gold NPs	15 nm (showed effective transport in both *in vitro* and *in vivo* mice model)	Ohta et al. (2020)
Gold NPs	5 nm or more. (proven efficient by Caveolar endocytosis *in vitro*)	Male et al. (2016)
Gold NPs	Demonstrated that 70 nm are optimal within the brain cells. 20 nm are optimal for free surface area	Shilo et al. (2015)
Insulin targeted – Gold NPs	20 nm (>5% of injected dose was reported to cross the BBB)	Shilo et al. (2014)
Silica NPs	100 nm Spheres	Hanada et al. (2014)
Silver NPs	50–100 nm (Can cross the BBB easily but leads to toxicity on prolonged accumulation)	Tang et al. (2008)
Solid Lipid NPs	60–125 nm (with different conjugations)	Agarwal et al. (2011), Lockman et al. (2003), Kadari et al. (2018)

## Side Effects of Nanoparticles Used for Neurotherapy

Despite the several theranostic applications, nanotechnologies still have many limitations, risk factors and side effects in their use particularly neural diagnosis and therapy. The general impediment in the treatment of many neurological disorders and injuries to the CNS is due to the low regenerative capacity of these systems.

Magnetic Carriers: As described in earlier section these are the most widely employed nano carriers due to the magnetoporation abilities which enable the drug delivery across the BBB. However, for stability, they undergo wide variety of processing among which the effectivity enhancement is done by the addition of Fe2 + and Fe3 + in the colloid. Notably higher amounts of iron is toxic to the brain and the cells start to undergo apoptosis (Petters et al., 2014; Yarjanli et al., 2017).

The coating material also plays an important role in delivery, however, these coatings with some methods were reported to be toxic. A silicon-coated nanocapsule is targeted cross the BBB by cell membrane translocation mechanism it was reported to slightly reverse the astrogliosis (Kong et al., 2012). It was also reported that Aminosilane or ethylenediaminetriacetate (EDT) coating with a hydro diameter of 25 or 29 nm when targeted through mannitol infusion resulted in an efficient influx, however, the permeability was observed to remain unchanged for a prolonged period which posed a risk of entry of several other non-permeable materials beyond the BBB (Sun et al., 2014). In another set of experiments conducted in rats, it was noted that IONs coated with Poly (maleic acid-co-olefin) with the temperature being the method of to open tight junctions of BBB resulted in the reversibility of opening and disrupted the brain immune responses (Tabatabaei et al., 2015). When loosening the junctions of BBB through thermotherapy, Alternating magnetic field (AMF) has to be taken care of as higher AMF leads to an increased temperature which could affect the cell viability (Dan et al., 2015).

Copper oxide nanoparticles (CuONPs) were also attested by several researchers to be potentially neurotoxic in nature where certain working concentrations elevated DNA fragmentation and cell death. In addition, these NPs were also demonstrated to inhibit voltage-gated sodium currents affecting the potentiation thus affecting learning and memory (Liu et al., 2011; An et al., 2012).

It has been observed that silver NPs (25 nm) could strongly disrupt the BBB by interacting with cerebral microvasculature and inducing proinflammatory cascades led to neurodegeneration and astrocyte reactivity (Trickler et al., 2010). In addition, silver NPs were also observed to increase inflammation, interfere with neuronal growth and differentiation and enhance amyloid precursor protein (APP) expression which ultimately leads to the formation of Aβ plaques (Lin et al., 2016).

The Silica NPs were observed to lower cellular density, decrease dendrite like processes and elevate Aβ1-42 peptide which is a hallmark for AD (Yang et al., 2014)

The nanoparticles, even at lower concentrations could lead to hazardous impacts in the context of neurodegenerative disorders like Parkinson’s disease and Alzheimer’s disease (Campbell, 2004). It has been recently highlighted that the neurotoxicity (in terms of altered genes, oxidative stress and neuroinflammation) was induced by engineered or combustion derived nanoparticles in animal models and in humans (Win-Shwe and Fujimaki, 2011; Bencsik et al., 2017). Hence it becomes indispensable 1) to review and comment on the influence of rapidly exposed nanoparticles on the CNS 2) to create awareness about the prevention of nanoparticle-induced neurodegenerative disorders like Parkinson’s disease and Alzheimer’s disease 3) to explore the possible treatment regime that could alleviate the neurotoxic effect of nanoparticles.

The size of the nanoparticle are so small and have greater surface area per unit mass, therefore, the probability of the relative number of atoms or functional groups exposed more to interact with the biological surface (Albanese et al.,2012; Crucho and Barros, 2017). Hence, the pharmacological effect and drug efficacy is more than conventional therapy. Constant popularity of nanoparticle-based therapy and widespread use and production of nanomaterials enhances the risk to the workers and consumers who are getting exposure directly. The nanoparticles have several adverse effects basing on the therapeutic materials and route of administration. The intensity of the penetration of the drug into the human body depends on the interactions between the type of nanoparticles and the exposing sites or organs such as lungs, gastrointestinal tract, mucosa and the skin. It can affect the respiratory epithelium as well as olfactory epithelium on inhalation (De Jong and Borm, 2008; De Matteis and Rinaldi, 2018). Further, the probability of penetration of the particles into the system may depend on their properties, affinities, point of contact and exposure time (Elder et al., 2006; Auría-Soro et al., 2019). The crucial stage for any drug in the treatment of neural diseases is to diffuse through BBB, and the targeted delivery of the drug into the CNS which makes the ultimate outcome against neurological diseases (Fonseca-Santos et al., 2015; Brzica et al., 2017).

## Toxicity Testing of Different Nanoparticles and Strategies to Reduce the Toxic Effects

The basic goals of the theranostic approach of treatment is to ensure the modalities of the therapeutic and diagnostic that develop specific and individualized therapeutic strategies towards personalized medicine (Funkhouser, 2002). However, the acceptable efficacy is very limited among the patients of specific treatment could be achieved for only very few patients. Therefore, new protocols could be adapted by combining therapeutic and diagnostic capability into one single agent to tailor a treatment based on the test results, thereby enhancing the mechanisms for curing the disease with more specific and efficient systems. The efficacy and success of such nanotheranostic agents depend on a number of inherent properties that lie in the nanoparticles. One of the major important characteristics of using NPs as theranostic agents is the probability to localize them in specific sites of diseases and diminish undesired side effects. Despite several advantages, few complications are also associated with a nanoparticle drug delivery system, and one of the major disadvantage is nanotoxicity of different nanoparticles. Nanoparticles can potentially show their toxicity as they have greater stability to retain in the human body for a prolonged period after inhaled, ingested or entered readily through the skin (Borm et al., 2006; Bergin and Witzmann, 2013). Once these materials entered into the body, they may be translocated into the entire system via blood circulation. The cause the easy spread into the entire body as they are small in size and for their surface characteristics such as polarity, hydrophilicity, lipophilicity and catalytic activity (Yang et al., 2016). The most common hazards from nanoparticles include neuroinflammation, oxidative stress, induced apoptosis and autophagy. These collectively affect the blood-brain barrier functions (Gao and Jiang, 2017).

It has been observed that the anionic nanoparticles are less toxic than the cationic particles like polystyrene and gold nanoparticles that cause hemolysis and clotting (De Jong et al., 2008). Researcher have assumed that the small size NPs might have more toxicity than their larger counterparts as they are less size with more surface area per unit mass. Moreover, the smaller NPs are easily taken up by the cells and exhibited more biochemical activity in the body than the larger size particles (Borm, et al., 2006; Dhawan and Sharma, 2010). Therefore, it is highly essential to evaluate their toxicity and health hazard effects.

Several possible routes are established for the entry of nanomaterials into the human body through dermal penetration, inhalation, ingestion, and systemic administration they may be accumulated in different tissues and organs including the brain (Burch, 2002). Different toxic and health hazard elements such as silver, gold, iron, copper, zinc, iron, cerium, manganese, titanium, aluminium, silica, and other carbon-based nanomaterials are used as NPs for various purposes in certain industries like automobile, electronics and communications, aerospace, chemical and paint, and pharmaceutical industries to which human exposure is very frequent and may cause several health-related problems including neurotoxicity (Persson et al., 2003; Sharma, 2009; Song and Tang, 2011). Metal oxide NPs have the capacity to translocate in a different part of the brain and can accumulate in olfactory bulb, cortex, and cerebellum via olfactory nerve pathway. Both *in vivo* and *in vitro* studies revealed the induction of elevated ROS levels due to toxicities of nanoparticles that can cause oxidative stress, inflammatory responses, and pathological changes.

Irrespective of their size, the amorphous TiO2 (30 nm) and silver nanoparticles (15 nm) induce the generation of free radicals, and may results in micronuclei formation by potentially damaging the genome. Moreover, macrophages mediated engulfment of the silver nanoparticles and quantum dots can also enhance the expression of inflammatory mediators like TNF-α, MIP-2 and IL-1β (Khanna et al., 2015). De Lorenzo (2008) reported on intranasal administration of silver-coated colloidal gold particles (50 nm) to the squirrel monkeys, the NPs showed anterogradely move from the axons of the olfactory nerve to the olfactory bulbs. It has also observed that on the exposure of manganese, nickel, cadmium, and cobalt nanomaterials via olfactory epithelium, they can translocate to the brain via olfactory neurons (Tjälve et al., 1996; Tjälve and Henriksson, 1999). In recent years, increased amount of environmental pollutants, including NPs have also been a crucial role for increasing the number of neurodegenerative diseases such as Alzheimer’s disease, Parkinson’s disease, Prion disease, amyotrophic lateral sclerosis or Huntington’s disease have been diagnosed and treated (Lanone and Boczkowski, 2006; Ai Tran et al., 2010; Bondì et al., 2010; Peters et al., 2011). Here, the role of BBB is critical in understanding NP toxicity as it disturbs the permeability properties of the BBB that modulates the extended plasma membrane that contains tight junctions between the adjacent endothelial cells of the cerebral capillaries along with the astrocytic endfeet cover (85%) and basement membrane that combinedly supports the BBB function (Henriksson and Tjalve, 2000; Sharma et al., 2011; Fonseca-Santos et al., 2015; Brzica et al., 2017). It has been shown that administration of Ag, Cu, or Al NPs (50–60 nm) by intravenous, intraperitoneal, or intracerebral route disrupts the BBB, as evident by staining with albumin-bound Evans blue (Ahmed and Gubrud, 2004). Nanoparticles can also stimulate the microenvironment of the vesicular transport system in order to gain access to the CNS and thereby exert a toxic effect in the CNS. Therefore, it is essential to have some robust strategies to cross the BBB without disturbing its microenvironment.

In two different *in vivo* studies of glioma, Au + Ni80Fe20 and Octadecyl-quaternized carboxymethyl chitosan were observed to prolong the survivability compared to untreated mice (Cheng et al., 2016). In another experiment, the use of Ferumoxide-labeled human neural stem cells resulted in stroke recovery without any toxic effects in rats (Song et al., 2015). In a recent study, the researchers have demonstrated that convection-enhanced delivery of iron oxide NPs were able to potential carry the payload for treating malignant glioma without causing cytotoxicity (Bernal et al., 2014). In another study magnetofection using Oleic acid-coated MNPs and Alpha-Synuclein RNAi Plasmid as genetic delivery target resulted in a significant motor improvement and reduced neurodegeneration in a Parkinson’s disease (PD) rats (Niu et al., 2017).

PLGA [poly (lactic-co-glycolic acid)] NPs of smaller size loaded with cerebrolysin and coupled to a CW800 imaging agent were demonstrated to reduce brain pathology following a TBI by effectively reducing the BBB breakdown caused due to the injury (Ruozi et al., 2015; Cruz et al., 2016). Liposomes containing both docetaxel and QDs when decorated with RGD-TGPS were shown to be more effective than the conventional drug and have demonstrated limited ROS generation (Sonali et al., 2016).

Recent, study based on the designing of the nanoparticles that reveal the strategies to interact the BBB cells at the molecular level, exploiting the existing physiological mechanisms of transport, without intervening with the normal function of the barrier itself. There are some encouraging mechanisms, Receptor- and Adsorptive-mediated transcytosis that facilitates transcellular transport of nanoparticles from the blood to the brain (Bhaskar et al., 2010). In Adsorptive-mediated transcytosis, the electrostatic interaction of a ligand with charges expressed at the luminal surface of endothelial cells. Cell-penetrating peptides (e.g. TAT-derived peptides) and cationic proteins (e.g. albumin) are commonly used on the surface of the nanomaterial that facilitate to cross the BBB (Bhaskar et al., 2010). On the other hand, Receptor-mediated transcytosis act upon the receptors expressed in BBB cells and exerts its selective drive to functionalize nanomaterial across the BBB endothelial cells. The common examples of this type are transferrin, insulin, apolipo-E which reaches the brain by this mechanism (Kim et al., 2007). Monoclonal antibodies are also used against the receptors present on the BBB as a bain-target drug delivery device. Monoclonal antibodies are also used against the receptors present on the BBB as a brain-target drug delivery device (Boado, 2008). The mAb raised for transferrin receptor (TfR) are 8D3, OX26 and R17217 may be used in this case to avoid the endogenous competition with transferrin in blood as they recognize different epitopes (Ulbrich et al., 2009). However, several issues are there to address when designing of this type of drug delivery system into the brain. The features of the nanomaterial should have minimum surface functionalization, prolonged half-line in blood, biodegradable and biocompatible, non-immunogenic, non-inflammatory and should be non-toxic.

## Conclusion

The present review concentrates on the different categories of nanoparticles with theranostic values. A discussion related to their cytotoxicity in the nervous system is highlighted. Certain regulatory guidelines for nanoparticles is the need of hour. Majority of the studies do not have well-defined pharmacokinetics and this establishment is crucial for understanding the half-life and other properties of several nanoparticles. The process or the mechanisms by which these theranostic agents cause the neural cytotoxicity is superficial. Therefore, the availability of standard toxicity testing assays could help invalidation of the NPS properties and their associated conjugates.

## References

[B1] Agarwal A. Majumder S. Agrawal H. Majumdar S. Agrawal P. G. (2011). Cationized albumin conjugated solid lipid nanoparticles as vectors for brain delivery of an anti-cancer drug. Curr. nano. 7, 71–40. 10.2174/157341311794480291

[B2] Aguilar Cosme J. R. Bryant H. E. Claeyssens F. (2019). Carbon dot-protoporphyrin IX conjugates for improved drug delivery and bioimaging. PLoS One 14, e0220210. 10.1371/journal.pone.0220210 31344086 PMC6657888

[B3] Ahmed J. Gubrud M. (2004). Anticipating military nanotechnology. Technol. Soc. Mag. IEEE 23, 33–40. 10.1109/MTAS.2004.1371637

[B4] Ai Tran H. N. A Sousa F. Moda F. Mandal S. Chanana M. Vimercati C. (2010). A novel class of potential prion drugs: preliminary *in vitro* and *in vivo* data for multilayer coated gold nanoparticles. Nanoscale 2, 2724–2732. 10.1039/c0nr00551g 20944860

[B5] Albanese A. Tang P. S. Chan W. C. (2012). The effect of nanoparticle size, shape, and surface chemistry on biological systems. Annu. Rev. Biomed. Eng. 14, 1–16. 10.1146/annurev-bioeng-071811-150124 22524388

[B6] An L. Liu S. Yang Z. Zhang T. (2012). Cognitive impairment in rats induced by nano-CuO and its possible mechanisms. Toxicol. Lett. 213, 220–227. 10.1016/j.toxlet.2012.07.007 22820425

[B7] Auría-Soro C. Nesma T. Juanes-Velasco P. Landeira-Viñuela A. Fidalgo-Gomez H. Acebes-Fernandez V. (2019). Interactions of nanoparticles and biosystems: microenvironment of nanoparticles and biomolecules in nanomedicine. Nanomater. (Basel). 9, 1365. 10.3390/nano9101365 PMC6835394 31554176

[B8] Bae K. H. Lee K. Kim C. Park T. G. (2011). Surface functionalized hollow manganese oxide nanoparticles for cancer targeted siRNA delivery and magnetic resonance imaging. Biomater. 32, 176–184. 10.1016/j.biomaterials.2010.09.039 20934746

[B9] Barua S. Yoo J. Kolhar P. Wakankar A. Gokarn Y. R. Mitragotri S. (2013). Particle shape enhances specificity of antibody-displaying nanoparticles. Proc. Natl. Acad. Sci. United States 110, 3270–3275. 10.1073/pnas.1216893110 PMC3587278 23401509

[B10] Bencsik A. Lestaevel P. Canu I. G. (2017). Nano- and neurotoxicology: an emerging discipline. Prog. Neurobiol. 160, 45–63. 10.1016/j.pneurobio.2017.10.003 29108800

[B11] Bergin I. L. Witzmann F. A. (2013). Nanoparticle toxicity by the gastrointestinal route: evidence and knowledge gaps. Int. J. Biomed. Nanosci. Nanotechnol. 3, 163–210. 10.1504/IJBNN.2013.054515 PMC3822607 24228068

[B12] Bernal G. M. LaRiviere M. J. Mansour N. Pytel P. Cahill K. E. Voce D. J. (2014). Convection-enhanced delivery and *in vivo* imaging of polymeric nanoparticles for the treatment of malignant glioma. Nanomedi. 10, 149–157. 10.1016/j.nano.2013.07.003 PMC3871979 23891990

[B13] Bhaskar S. Tian F. Stoeger T. Kreyling W. de la Fuente J. M. Grazú V. (2010). Multifunctional nanocarriers for diagnostics, drug delivery and targeted treatment across blood-brain barrier: perspectives on tracking and neuroimaging. Part. Fibre. Toxicol. 7, 3–25. 10.1186/1743-8977-7-3 20199661 PMC2847536

[B14] Blanco E. Shen H. Ferrari M. (2015). Principles of nanoparticle design for overcoming biological barriers to drug delivery. Nat. Biotechnol. 33, 941–951. 10.1038/nbt.3330 26348965 PMC4978509

[B15] Boado R. J. (2008). A new generation of neurobiological drugs engineered to overcome the challenges of brain drug delivery. Drug News Perspect. 21, 489–503. 10.1358/dnp.2008.21.9.1290820 19180267

[B16] Bondì M. L. Craparo E. F. Giammona G. Drago F. (2010). Brain-targeted solid lipid nanoparticles containing riluzole: preparation, characterization and biodistribution. Nanomedicine (Lond) 5, 25–32. 10.2217/nnm.09.67 20025461

[B17] Borm P. J. Robbins D. Haubold S. Kuhlbusch T. Fissan H. Donaldson K. (2006). The potential risks of nanomaterials: a review carried out for ECETOC. Part. Fibre. Toxicol. 3, 11. 10.1186/1743-8977-3-11 16907977 PMC1584248

[B18] Brzica H. Abdullahi W. Ibbotson K. Ronaldson P. T. (2017). Role of transporters in central nervous system drug delivery and blood-brain barrier protection: relevance to treatment of stroke. J. Cent. Nerv. Syst. Dis. 9, 1179573517693802. 10.1177/1179573517693802 28469523 PMC5392046

[B19] Burch W. M. (2002). Passage of inhaled particles into the blood circulation in humans. Circulation 106, e141–e142. 10.1161/01.cir.0000037134.24080.42 12427664

[B20] Campbell A. (2004). Inflammation, neurodegenerative diseases, and environmental exposures. Ann. N. Y Acad. Sci. 1035, 117–132. 10.1196/annals.1332.008 15681804

[B21] Ceña V. Játiva P. (2018). Nanoparticle crossing of blood-brain barrier: a road to new therapeutic approaches to central nervous system diseases. Nanomedicine (Lond) 13, 1513–1516. 10.2217/nnm-2018-0139 29998779

[B22] Chen R. Romero G. Christiansen M. G. Mohr A. Anikeeva P. (2015). Wireless magnetothermal deep brain stimulation. Science. 347, 1477–1480. 10.1126/science.1261821 25765068

[B23] Cheng Y. Dai Q. Morshed R. A. Fan X. Wegscheid M. L. Wainwright D. A. (2014). Blood-brain barrier permeable gold nanoparticles: an efficient delivery platform for enhanced malignant glioma therapy and imaging. Small. 10, 5137–5150. 10.1002/smll.201400654 25104165 PMC4268041

[B24] Cheng Y. Muroski M. E. Petit D. C. M. C. Mansell R. Vemulkar T. Morshed R. A. (2016). Rotating magnetic field induced oscillation of magnetic particles for *in vivo* mechanical destruction of malignant glioma. J. Control. Release 223, 75–84. 10.1016/j.jconrel.2015.12.028 26708022 PMC4724455

[B25] Chenthamara D. Subramaniam S. Ramakrishnan S. G. Krishnaswamy S. Essa M. M. Lin F. (2019). Therapeutic efficacy of nanoparticles and routes of administration. Biomater. Res. 23, 1–29. 10.1186/s40824-019-0166-x 31832232 PMC6869321

[B26] Chertok B. David A. E. Huang Y. Yang V. C. (2007). Glioma selectivity of magnetically targeted nanoparticles: a role of abnormal tumor hydrodynamics. J. Control Release. 122, 315–323. 10.1016/j.jconrel.2007.05.030 17628157 PMC2094531

[B27] Cooper J. M. Wiklander P. B. Nordin J. Z. Al-Shawi R. Wood M. J. Vithlani M. (2014). Systemic exosomal siRNA delivery reduced alpha-synuclein aggregates in brains of transgenic mice. Mov. Disord. 29, 1476–1485. 10.1002/mds.25978 25112864 PMC4204174

[B28] Costa P. M. Wang J. T. Morfin J. F. Khanum T. To W. Sosabowski J. (2018). Functionalised carbon nanotubes enhance brain delivery of amyloid-targeting pittsburgh compound B (PiB)-derived ligands. Nanothera. 2, 168–183. 10.7150/ntno.23125 PMC5865270 29577020

[B29] Crucho C. I. C. Barros M. T. (2017). Polymeric nanoparticles: a study on the preparation variables and characterization methods. Mater. Sci. Eng. C. Mater. Biol. Appl. 80, 771–784. 10.1016/j.msec.2017.06.004 28866227

[B30] Cruz L. J. Stammes M. A. Que I. van Beek E. R. Knol-Blankevoort V. T. Snoeks T. J. A. (2016). Effect of PLGA NP size on efficiency to target traumatic brain injury. J. Control Release 223, 31–41. 10.1016/j.jconrel.2015.12.029 26708021

[B31] Dan M. Bae Y. Pittman T. A. Yokel R. A. (2015). Alternating magnetic field-induced hyperthermia increases iron oxide nanoparticle cell association/uptake and flux in blood-brain barrier models. Pharm. Res. 32, 1615–1625. 10.1007/s11095-014-1561-6 25377069 PMC4803069

[B32] De Jong W. H. Borm P. J. (2008). Drug delivery and nanoparticles:applications and hazards. Int. J. Nanomedi. 3, 133–149. 10.2147/ijn.s596 PMC2527668 18686775

[B33] De Jong W. H. Hagens W. I. Krystek P. Burger M. C. Sips A. J. Geertsma R. E. (2008). Particle size-dependent organ distribution of gold nanoparticles after intravenous administration. Biomater. 29, 1912–1919. 10.1016/j.biomaterials.2007.12.037 18242692

[B34] De Lorenzo A. J. D. (2008). “The olfactory neuron and the blood brain barrier,” in Ciba Foundation symposium e internal secretions of the pancreas (colloquia on endocrinology) (Hoboken, NJ, United States: John Wiley & Sons), 151–176.

[B35] De Matteis V. Rinaldi R. (2018). Toxicity assessment in the nanoparticle era. Adv. Exp. Med. Biol. 1048, 1–19. 10.1007/978-3-319-72041-8_1 29453529

[B36] Dhawan A. Sharma V. (2010). Toxicity assessment of nanomaterials: methods and challenges. Anal. Bioanal. Chem. 398, 589–605. 10.1007/s00216-010-3996-x 20652549

[B37] Elder A. Gelein R. Silva V. Feikert T. Opanashuk L. Carter J. (2006). Translocation of inhaled ultrafine manganese oxide particles to the central nervous system. Environ. Health Perspect. 114, 1172–1178. 10.1289/ehp.9030 16882521 PMC1552007

[B38] Faustino C. Rijo P. Reis C. P. (2017). Nanotechnological strategies for nerve growth factor delivery: therapeutic implications in Alzheimer's disease. Pharmacol. Res. 120, 68–87. 10.1016/j.phrs.2017.03.020 28351757

[B39] Fonseca-Santos B. Gremião M. P. Chorilli M. (2015). Nanotechnology-based drug delivery systems for the treatment of Alzheimer's disease. Int. J. Nanomedi. 10, 4981–5003. 10.2147/IJN.S87148 PMC4531021 26345528

[B40] Funkhouser J. (2002). Reinventing pharma: the theranostic revolution. Curr. Drug Discov. 2, 17–19.

[B41] Fuster-Matanzo A. Gessler F. Leonardi T. Iraci N. Pluchino S. (2015). Acellular approaches for regenerative medicine: on the verge of clinical trials with extracellular membrane vesicles?. Stem Cel Res. Ther. 6, 227. 10.1186/s13287-015-0232-9 PMC4668616 26631254

[B42] Gao H. Jiang X. (2017). “Introduction and overview,” in Neurotoxicity of nanomaterials and nanomedicine. Editor Jiang X. Gao H. (Cambridge, MA: Academic Press), 1–31.

[B43] Gao J. Gu H. Xu B. (2009). Multifunctional magnetic nanoparticles: design, synthesis, and biomedical applications. Acc. Chem. Res. 42, 1097–1107. 10.1021/ar9000026 19476332

[B44] Guiot C. Zullino S. Priano L. Cavalli R. (2016). The physics of drug-delivery across the blood-brain barrier. Ther. Deliv. 7, 153–156. 10.4155/tde-2016-0001 26893246

[B45] Hanada S. Fujioka K. Inoue Y. Kanaya F. Manome Y. Yamamoto K. (2014). Cell-based *in vitro* blood-brain barrier model can rapidly evaluate nanoparticles' brain permeability in association with particle size and surface modification. Int. J. Mol. Sci. 15, 1812–1825. 10.3390/ijms15021812 24469316 PMC3958822

[B46] Henriksson J. Tjälve H. (2000). Manganese taken up into the CNS via the olfactory pathway in rats affects astrocytes. Toxicol. Sci. 55, 392–398. 10.1093/toxsci/55.2.392 10828272

[B122] Huang P. Bao L. Zhang C. Lin J. Luo T. Yang D. (2011). Folic acid-conjugated silica-modified gold nanorods for X-ray/CT imaging-guided dual-mode radiation and photo-thermal therapy. Biomaterials 32, 9796–9809. 10.1016/j.biomaterials.2011.08.086 21917309

[B47] Jain T. K. Richey J. Strand M. Leslie-Pelecky D. L. Flask C. A. Labhasetwar V. (2008). Magnetic nanoparticles with dual functional properties: drug delivery and magnetic resonance imaging. Biomater. 29, 4012–4021. 10.1016/j.biomaterials.2008.07.004 PMC2593647 18649936

[B48] Kadari A. Pooja D. Gora R. H. Gudem S. Kolapalli V. R. M. Kulhari H. (2018). Design of multifunctional peptide collaborated and docetaxel loaded lipid nanoparticles for antiglioma therapy. Eur. J. Pharm. Biopharm. 132, 168–179. 10.1016/j.ejpb.2018.09.012 30244167

[B49] Karthivashan G. Ganesan P. Park S. Y. Kim J. S. Choi D. K. (2018). Therapeutic strategies and nano-drug delivery applications in management of ageing Alzheimer's disease. Drug Deliv. 25, 307–320. 10.1080/10717544.2018.1428243 29350055 PMC6058502

[B50] Khanna P. Ong C. Bay B. H. Baeg G. H. (2015). Nanotoxicity: an interplay of oxidative stress, inflammation and cell death. Nanomater. (Basel). 5, 1163–1180. 10.3390/nano5031163 PMC5304638 28347058

[B51] Kim H. R. Andrieux K. Gil S. Taverna M. Chacun H. Desmaële D. (2007). Translocation of poly(ethylene glycol-co-hexadecyl)cyanoacrylate nanoparticles into rat brain endothelial cells: role of apolipoproteins in receptor-mediated endocytosis. Biomacromol. 8, 793–799. 10.1021/bm060711a 17309294

[B52] Kohler N. Sun C. Wang J. Zhang M. (2005). Methotrexate-modified superparamagnetic nanoparticles and their intracellular uptake into human cancer cells. Langmuir. 21, 8858–8864. 10.1021/la0503451 16142971

[B53] Kolhar P. Anselmo A. C. Gupta V. Pant K. Prabhakarpandian B. Ruoslahti E. (2013). Using shape effects to target antibody-coated nanoparticles to lung and brain endothelium. Proc. Natl. Acad. Sci. United States 110, 10753–10758. 10.1073/pnas.1308345110 PMC3696781 23754411

[B54] Kong S. D. Lee J. Ramachandran S. Eliceiri B. P. Shubayev V. I. Lal R. (2012). Magnetic targeting of nanoparticles across the intact blood-brain barrier. J. Control Release. 164, 49–57. 10.1016/j.jconrel.2012.09.021 23063548 PMC4440873

[B55] Krishna V. Singh A. Sharma P. Iwakuma N. Wang Q. Zhang Q. (2010). Polyhydroxy fullerenes for non-invasive cancer imaging and therapy. Small. 6, 2236–2241. 10.1002/smll.201000847 20818623

[B56] Lakshmanan S. Gupta G. K. Avci P. Chandran R. Sadasivam M. Jorge A. E. (2014). Physical energy for drug delivery; poration, concentration and activation. Adv. Drug Deliv. Rev. 71, 98–114. 10.1016/j.addr.2013.05.010 23751778 PMC3849347

[B57] Lanone S. Boczkowski J. (2006). Biomedical applications and potential health risks of nanomaterials: molecular mechanisms. Curr. Mol. Med. 6, 651–663. 10.2174/156652406778195026 17022735

[B58] Lee J. H. Huh Y. M. Jun Y. W. Seo J. W. Jang J. T. Song H. T. (2007). Artificially engineered magnetic nanoparticles for ultra-sensitive molecular imaging. Nat. Med. 13, 95–99. 10.1038/nm1467 17187073

[B121] Lee J. H. Lee K. Moon S. H. Lee Y. Park T. G. Cheon J. (2009). All-in-one target-cell-specific magnetic nanoparticles for simultaneous molecular imaging and siRNA delivery. Angew. Chem. Int. Ed Engl. 48, 4174–4179. 10.1002/anie.200805998 19408274

[B59] Li Z. Chen H. Bao H. Gao M. (2004). One-pot reaction to synthesize water-soluble magnetite nanocrystals. Chem. Mater. 16, 1391–1393. 10.1021/cm035346y

[B60] Lin H. C. Huang C. L. Huang Y. J. Hsiao I. L. Yang C. W. Chuang C. Y. (2016). Transcriptomic gene-network analysis of exposure to silver nanoparticle reveals potentially neurodegenerative progression in mouse brain neural cells. Toxicol. Vitro. 34, 289–299. 10.1016/j.tiv.2016.04.014 27131904

[B61] Liu Z. Liu S. Ren G. Zhang T. Yang Z. (2011). Nano-CuO inhibited voltage-gated sodium current of hippocampal CA1 neurons via reactive oxygen species but independent from G-proteins pathway. J. Appl. Toxicol. 31, 439–445. 10.1002/jat.1611 21218498

[B62] Lockman P. R. Oyewumi M. O. Koziara J. M. Roder K. E. Mumper R. J. Allen D. D. (2003). Brain uptake of thiamine-coated nanoparticles. J. Control. Release. 93, 271–282. 10.1016/j.jconrel.2003.08.006 14644577

[B63] Lu S. Guo S. Xu P. Li X. Zhao Y. Gu W. (2016). Hydrothermal synthesis of nitrogen-doped carbon dots with real-time live-cell imaging and blood-brain barrier penetration capabilities. Int. J. Nanomedicine 11, 6325–6336. 10.2147/IJN.S119252 27932880 PMC5135288

[B64] Male D. Gromnicova R. McQuaid C. (2016). Gold nanoparticles for imaging and drug transport to the CNS. Int. Rev. Neurobiol. 130, 155–198. 10.1016/bs.irn.2016.05.003 27678177

[B65] Martins S. Tho I. Reimold I. Fricker G. Souto E. Ferreira D. (2012). Brain delivery of camptothecin by means of solid lipid nanoparticles: formulation design, *in vitro* and *in vivo* studies. Int. J. Pharm. 439, 49–62. 10.1016/j.ijpharm.2012.09.054 23046667

[B66] Matea C. T. Mocan T. Tabaran F. Pop T. Mosteanu O. Puia C. (2017). Quantum dots in imaging, drug delivery and sensor applications. Int. J. Nanomedicine. 12, 5421–5431. 10.2147/IJN.S138624 28814860 PMC5546783

[B67] McDonagh B. H. Singh G. Hak S. Bandyopadhyay S. Augestad I. L. Peddis D. (2016). L-DOPA-Coated manganese oxide nanoparticles as dual MRI contrast agents and drug-delivery vehicles. Small. 12, 301–306. 10.1002/smll.201502545 26619158

[B68] Mo J. He L. Ma B. Chen T. (2016). Tailoring particle size of mesoporous silica nanosystem to antagonize glioblastoma and overcome blood-brain barrier. ACS Appl. Mater. Inter. 8, 6811–6825. 10.1021/acsami.5b11730 26911360

[B69] Morel A. L. Nikitenko S. I. Gionnet K. Wattiaux A. Lai-Kee-Him J. Labrugere C. (2008). Sonochemical approach to the synthesis of Fe(3)O(4)@SiO(2) core-shell nanoparticles with tunable properties. ACS Nano. 2, 847–856. 10.1021/nn800091q 19206481

[B70] Munshi R. Qadri S. M. Zhang Q. Castellanos Rubio I. del Pino P. Pralle A. (2017). Magnetothermal genetic deep brain stimulation of motor behaviors in awake, freely moving mice. Elife 6, e27069. 10.7554/eLife.27069 28826470 PMC5779110

[B71] Nance E. Zhang F. Mishra M. K. Zhang Z. Kambhampati S. P. Kannan R. M. (2016). Nanoscale effects in dendrimer-mediated targeting of neuroinflammation. Biomater. 101, 96–107. 10.1016/j.biomaterials.2016.05.044 PMC5379995 27267631

[B72] Niu S. Zhang L. K. Zhang L. Zhuang S. Zhan X. Chen W. Y. (2017). Inhibition by multifunctional magnetic nanoparticles loaded with alpha-synuclein RNAi plasmid in a Parkinson's disease model. Theranostics 7, 344–356. 10.7150/thno.16562 28042339 PMC5197069

[B73] Nowak M. Brown T. D. Graham A. Helgeson M. E. Mitragotri S. (2020). Size, shape, and flexibility influence nanoparticle transport across brain endothelium under flow Bioeng. Transl Med. 5, e10153. 10.1002/btm2.10153 32440560 PMC7237148

[B74] Ohta S. Kikuchi E. Ishijima A. Azuma T. Sakuma I. Ito T. (2020). Investigating the optimum size of nanoparticles for their delivery into the brain assisted by focused ultrasound-induced blood-brain barrier opening. Sci. Rep. 10, 18220. 10.1038/s41598-020-75253-9 33106562 PMC7588485

[B75] Pardo M. Roberts E. R. Pimentel K. Yildirim Y. A. Navarrete B. Wang P. (2020). Size-dependent intranasal administration of magnetoelectric nanoparticles for targeted brain localization. Nanomedi. 32, 102337. 10.1016/j.nano.2020.102337 33197627

[B76] Paris-Robidas S. Brouard D. Emond V. Parent M. Calon F. (2016). Internalization of targeted quantum dots by brain capillary endothelial cells *in vivo* . J. Cereb. Blood Flow Metab. 36, 731–742. 10.1177/0271678X15608201 26661181 PMC4820005

[B77] Perets N. Betzer O. Shapira R. Brenstein S. Angel A. Sadan T. (2019). Golden exosomes selectively target brain pathologies in neurodegenerative and neurodevelopmental disorders. Nano Lett. 19, 3422–3431. 10.1021/acs.nanolett.8b04148 30761901

[B78] Persson E. Henriksson J. Tjälve H. (2003). Uptake of cobalt from the nasal mucosa into the brain via olfactory pathways in rats. Toxicol. Lett. 145, 19–27. 10.1016/s0378-4274(03)00266-2 12962970

[B79] Peters A. Rückerl R. Cyrys J. (2011). Lessons from air pollution epidemiology for studies of engineered nanomaterials. J. Occup. Environ. Med. 53, S8–S13. 10.1097/JOM.0b013e31821ad5c0 21654423

[B80] Petters C. Irrsack E. Koch M. Dringen R. (2014). Uptake and metabolism of iron oxide nanoparticles in brain cells. Neurochem. Res. 39, 1648–1660. 10.1007/s11064-014-1380-5 25011394

[B81] Reddy M. K. Labhasetwar V. (2009). Nanoparticle-mediated delivery of superoxide dismutase to the brain: an effective strategy to reduce ischemia-reperfusion injury. FASEB J. 23, 1384–1395. 10.1096/fj.08-116947 19124559

[B82] Roy Chowdhury S. Mondal S. Muthuraj B. Balaji S. N. Trivedi V. Krishnan Iyer P. (2018). Remarkably efficient blood-brain barrier crossing polyfluorene-chitosan nanoparticle selectively tweaks amyloid oligomer in cerebrospinal fluid and aβ1-40. ACS Omega. 3, 8059–8066. 10.1021/acsomega.8b00764 30087934 PMC6072248

[B83] Ruozi B. Belletti D. Sharma H. S. Sharma A. Muresanu D. F. Mössler H. (2015). PLGA nanoparticles loaded cerebrolysin: studies on their preparation and investigation of the effect of storage and serum stability with reference to traumatic brain injury. Mol. Neurobiol. 52, 899–912. 10.1007/s12035-015-9235-x 26108180

[B84] Saesoo S. Sathornsumetee S. Anekwiang P. Treetidnipa C. Thuwajit P. Bunthot S. (2018). Characterization of liposome-containing SPIONs conjugated with anti-CD20 developed as a novel theranostic agent for central nervous system lymphoma. Colloids Surf. B Biointer. 161, 497–507. 10.1016/j.colsurfb.2017.11.003 29128836

[B85] Sharma H. S. (2009). A special section on nanoneuroscience: nanoneurotoxicity and nanoneuroprotection. J. Nanosci. Nanotechnol. 9, 4992–4995. 10.1166/jnn.2009.gr01 19928179

[B86] Sharma H. S. Muresanu D. F. Patnaik R. Stan A. D. Vacaras V. Perju-Dumbrav L. (2011). Superior neuroprotective effects of cerebrolysin in heat stroke following chronic intoxication of Cu or Ag engineered nanoparticles. A comparative study with other neuroprotective agents using biochemical and morphological approaches in the rat. J. Nanosci Nanotechnol 11, 7549–7569. 10.1166/jnn.2011.5114 22097459

[B87] Shilo M. Motiei M. Hana P. Popovtzer R. (2014). Transport of nanoparticles through the blood-brain barrier for imaging and therapeutic applications. Nanoscale 6, 2146–2152. 10.1039/c3nr04878k 24362586

[B88] Shilo M. Sharon A. Baranes K. Motiei M. Lellouche J. P. Popovtzer R. (2015). The effect of nanoparticle size on the probability to cross the blood-brain barrier: an in-vitro endothelial cell model. J. Nanobiotech. 13, 19. 10.1186/s12951-015-0075-7 PMC4359781 25880565

[B89] Singhal A. Morris V. B. Labhasetwar V. Ghorpade A. (2013). Nanoparticle-mediated catalase delivery protects human neurons from oxidative stress. Cell Death Dis. 4, e903. 10.1038/cddis.2013.362 24201802 PMC3847304

[B90] Sokolova V. Mekky G. van der Meer S. B. Seeds M. C. Atala A. J. Epple M. (2020). Transport of ultrasmall gold nanoparticles (2 nm) across the blood-brain barrier in a six-cell brain spheroid model. Sci. Rep. 10, 18033. 10.1038/s41598-020-75125-2 33093563 PMC7581805

[B91] Solanki A. Kim J. D. Lee K. (2008). Nanotechnology for regenerative medicine: nanomaterials for stem cell imaging. Nanomedicine (Lond) 3, 567–578. 10.2217/17435889.3.4.567 18694318

[B92] Sonali. Singh R. P. Sharma G. Kumari L. Koch B. Singh S. (2016). RGD-TPGS decorated theranostic liposomes for brain targeted delivery. Colloids Surf. B Biointerfaces 147, 129–141. 10.1016/j.colsurfb.2016.07.058 27497076

[B93] Song M. Kim Y. J. Kim Y. H. Roh J. Kim E. C. Lee H. J. (2015). Long-term effects of magnetically targeted ferumoxide-labeled human neural stem cells in focal cerebral ischemia. Cell. Transpl. 24, 183–190. 10.3727/096368913X675755 24380414

[B94] Song Y. Du D. Li L. Xu J. Dutta P. Lin Y. (2017). *In Vitro* study of receptor-mediated silica nanoparticles delivery across blood-brain barrier. ACS Appl. Mater. Inter. 9, 20410–20416. 10.1021/acsami.7b03504 PMC5533093 28541655

[B95] Song Y. Tang S. (2011). Nanoexposure, unusual diseases, and new health and safety concerns. Sci. Worl. J. 11, 1821–1828. 10.1100/2011/794801 PMC3201677 22125440

[B96] Souza K. C. Salazar-Alvarez G. Ardisson J. D. Macedo W. A. Sousa E. M. (2008). Mesoporous silica-magnetite nanocomposite synthesized by using a neutral surfactant. Nanotech. 19, 185603. 10.1088/0957-4484/19/18/185603 21825691

[B97] Spinelli A. Girelli M. Arosio D. Polito L. Podini P. Martino G. (2019). Intracisternal delivery of PEG-coated gold nanoparticles results in high brain penetrance and long-lasting stability. J. Nanobiotech. 17, 49. 10.1186/s12951-019-0481-3 PMC6448280 30943991

[B98] Sun Z. Worden M. Wroczynskyj Y. Yathindranath V. van Lierop J. Hegmann T. (2014). Magnetic field enhanced convective diffusion of iron oxide nanoparticles in an osmotically disrupted cell culture model of the blood-brain barrier. Int. J. Nanomed. 9, 3013–3026. 10.2147/IJN.S62260 PMC4073976 25018630

[B99] Tabatabaei S. N. Girouard H. Carret A. S. Martel S. (2015). Remote control of the permeability of the blood-brain barrier by magnetic heating of nanoparticles: a proof of concept for brain drug delivery. J. Control Release. 206, 49–57. 10.1016/j.jconrel.2015.02.027 25724273

[B100] Tang J. Xiong L. Wang S. Wang J. Liu L. Li J. (2008). Influence of silver nanoparticles on neurons and blood-brain barrier via subcutaneous injection in rats. Appl. Surf. Sci. 255, 502–504. 10.1016/j.apsusc.2008.06.058

[B101] Tay A. Di Carlo D. (2017). Remote neural stimulation using magnetic nanoparticles. Curr. Med. Chem. 24, 537–548. 10.2174/0929867323666160814000442 27528057

[B102] Tjälve H. Henriksson J. Tallkvist J. Larsson B. S. Lindquist N. G. (1996). Uptake of manganese and cadmium from the nasal mucosa into the central nervous system via olfactory pathways in rats. Pharmacol. Toxicol. 79, 347–356. 10.1111/j.1600-0773.1996.tb00021.x 9000264

[B103] Tjälve H. Henriksson J. (1999). Uptake of metals in the brain via olfactory pathways. Neurotoxicology 20, 181–195. 10385882

[B104] Tomitaka A. Arami H. Gandhi S. Krishnan K. M. (2015). Lactoferrin conjugated iron oxide nanoparticles for targeting brain glioma cells in magnetic particle imaging. Nanoscale 7, 16890–16898. 10.1039/c5nr02831k 26412614 PMC4626448

[B105] Trickler W. J. Lantz S. M. Murdock R. C. Schrand A. M. Robinson B. L. Newport G. D. (2010). Silver nanoparticle induced blood-brain barrier inflammation and increased permeability in primary rat brain microvessel endothelial cells. Toxicol. Sci. 118, 160–170. 10.1093/toxsci/kfq244 20713472

[B106] Turan O. Bielecki P. Perera V. Lorkowski M. Covarrubias G. Tong K. (2019). Delivery of drugs into brain tumors using multicomponent silica nanoparticles. Nanoscale. 11, 11910–11921. 10.1039/c9nr02876e 31187845 PMC7776621

[B107] Ulbrich K. Hekmatara T. Herbert E. Kreuter J. (2009). Transferrin- and transferrin-receptor-antibody-modified nanoparticles enable drug delivery across the blood-brain barrier (BBB). Eur. J. Pharm. Biopharm. 71, 251–256. 10.1016/j.ejpb.2008.08.021 18805484

[B108] Umarao P. Bose S. Bhattacharyya S. Kumar A. Jain S. (2016). Neuroprotective potential of superparamagnetic iron oxide nanoparticles along with exposure to electromagnetic field in 6-OHDA rat model of Parkinson's disease. J. Nanosci. Nanotechnol. 16, 261–269. 10.1166/jnn.2016.11103 27398453

[B109] Veiseh O. Kievit F. M. Mok H. Ayesh J. Clark C. Fang C. (2011). Cell transcytosing poly-arginine coated magnetic nanovector for safe and effective siRNA delivery. Biomaterials 32, 5717–5725. 10.1016/j.biomaterials.2011.04.039 21570721 PMC3125526

[B110] Voigt N. Henrich-Noack P. Kockentiedt S. Hintz W. Tomas J. Sabel B. A. (2014). Surfactants, not size or zeta-potential influence blood-brain barrier passage of polymeric nanoparticles. Eur. J. Pharm. Biopharm. 87, 19–29. 10.1016/j.ejpb.2014.02.013 24607790

[B111] Warner S. (2004). Diagnostics plus therapy = theranostics. Scientist 18, 38–39.

[B112] Win-Shwe T. T. Fujimaki H. (2011). Nanoparticles and neurotoxicity. Int. J. Mol. Sci. 12, 6267–6280. 10.3390/ijms12096267 22016657 PMC3189781

[B113] WHO (2006). Neurological disorders: public health challenges. (Geneva, Switzerland: WHO Press).

[B114] Xu H. Cheng L. Wang C. Ma X. Li Y. Liu Z. (2011). Polymer encapsulated upconversion nanoparticle/iron oxide nanocomposites for multimodal imaging and magnetic targeted drug delivery. Biomater. 32, 9364–9373. 10.1016/j.biomaterials.2011.08.053 21880364

[B115] Yang C. Tian A. Li Z. (2016). Reversible cardiac hypertrophy induced by PEG-coated gold nanoparticles in mice. Sci. Rep. 6, 20203–20212. 10.1038/srep20203 26830764 PMC4735330

[B116] Yang K. Hu L. Ma X. Ye S. Cheng L. Shi X. (2012). Multimodal imaging guided photothermal therapy using functionalized graphene nanosheets anchored with magnetic nanoparticles. Adv. Mater. Weinheim. 24, 1868–1872. 10.1002/adma.201104964 22378564

[B117] Yang X. He C. Li J. Chen H. Ma Q. Sui X. (2014). Uptake of silica nanoparticles: neurotoxicity and alzheimer-like pathology in human SK-N-SH and mouse neuro2a neuroblastoma cells. Toxicol. Lett. 229, 240–249. 10.1016/j.toxlet.2014.05.009 24831964

[B118] Yang X. Hong H. Grailer J. J. Rowland I. J. Javadi A. Hurley S. A. (2011). cRGD-functionalized, DOX-conjugated, and ⁶⁴Cu-labeled superparamagnetic iron oxide nanoparticles for targeted anticancer drug delivery and PET/MR imaging. Biomater. 32, 4151–4160. 10.1016/j.biomaterials.2011.02.006 PMC3292876 21367450

[B119] Yarjanli Z. Ghaedi K. Esmaeili A. Rahgozar S. Zarrabi A. (2017). Iron oxide nanoparticles may damage to the neural tissue through iron accumulation, oxidative stress, and protein aggregation. BMC Neurosci. 18, 51. 10.1186/s12868-017-0369-9 28651647 PMC5485499

[B120] Zhuang X. Xiang X. Grizzle W. Sun D. Zhang S. Axtell R. C. (2011). Treatment of brain inflammatory diseases by delivering exosome encapsulated anti-inflammatory drugs from the nasal region to the brain. Mol. Ther. 19, 1769–1779. 10.1038/mt.2011.164 21915101 PMC3188748

